# Circulating Coxsackievirus A16 Identified as Recombinant Type A Human Enterovirus, China

**DOI:** 10.3201/eid1708.101719

**Published:** 2011-08

**Authors:** Ke Zhao, Xue Han, Guanjun Wang, Wei Hu, Wenyan Zhang, Xiao-Fang Yu

**Affiliations:** Author affiliations: First Hospital of Jilin University, Changchun, People’s Republic of China (K. Zhao, X. Han, G. Wang, W. Hu, W. Zhang, X.-F. Yu);; Johns Hopkins University, Baltimore, Maryland, USA (K. Zhao, X.-F. Yu)

**Keywords:** hand, foot, and mouth disease, HFMD, enteroviruses, coxsackievirus, coxsackievirus A16, China, human enterovirus 71, viruses, dispatch, recombination

## Abstract

To determine the relationship of coxsackievirus A16 (CA16) to prototype CA16-G10, we conducted a phylogenetic analysis of circulating CA16 strains in China. Complex recombinant forms of CA16-related viruses involving multiple human enteroviruses, subgroup A (CA4, CA16, and enterovirus 71), are prevalent among patients with hand, foot, and mouth disease.

Coxsackievirus A16 (CA16) is a member of the family *Picornaviridae,* genus *Human enterovirus* (HEV). These viruses can be further divided into 4 subgroups on the basis of molecular typing: HEV-A, HEV-B, HEV-C, and HEV-D. The first, and prototype, CA16 strain, CA16-G10, was isolated in South Africa almost 60 years ago (*1*) and was subsequently sequenced in 1994 (*2*). CA16, along with enterovirus71 (EV71), CA2, and CA4, is a member of the HEV-A subgroup. CA16 is commonly associated with hand, foot, and mouth disease (HFMD) in children and sometimes causes aseptic meningitis, encephalitis, myocarditis, and poliomyelitis-like paralysis (*3*).

Enteroviruses related to HFMD have been endemic to Southeast Asia and the Pacific region for decades (*4**–**7*). Recently, a dramatic increase in HFMD prevalence has been reported in the People’s Republic of China (*8**–**10*). Partial viral sequencing (e.g., of the viral protein [VP] 1 region), serologic characterization, or both, have shown that 10%–50% of viruses from HFMD patients are related to prototype CA16-G10, and thus they have been classified as CA16 strains (*11*). The relationship of circulating CA16-related viruses to CA16-G10 has not been well studied. Therefore, we conducted a serial phylogenetic analysis of existing and new CA16 sequences from northern, central, and southern China to examine whether CA16–G10 truly is the parental strain of circulating CA16 strains. As a result, we found that current CA16 strains in China, although still related to CA16–G10, are recombinant HEV-A.

## The Study

**Figure 1:**
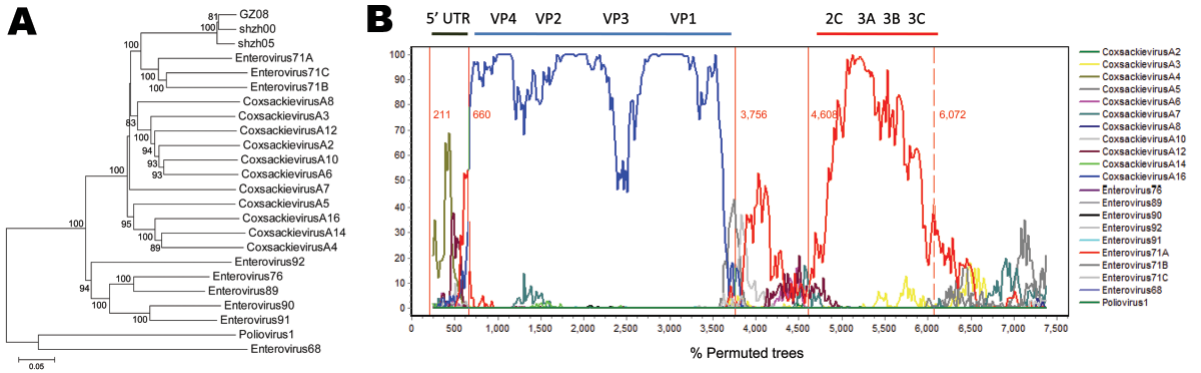
Classification of coxsackievirus 16A (CA16) sequences from the People’s Republic of China into human enterovirus (HEV) groups. A) Phylogenetic analysis performed by using all HEV reference samples from China classified as HEV-A but not as CA16. All sequences were retrieved from GenBank. The sequences used corresponded to positions 2–7,407 bp in shzh00-1. MEGA4 software (*12*) was used as the analytic program and Kimura 2-parameter as the model. The phylogenetic tree was determined for 1,000 replicates with random seeds. Only strong bootstrap values (>70%) are shown. Scale bar shows nucleotide substitutions per site. B) Bootscanning analysis of shzh00-1. For all HEV-A sequences, together with sequences from 2 outgroups, shzh00-1 showed possible recombination with CA4 and enterovirus 71A. The vertical red lines with numbers show the possible recombination break points as determined by genetic algorithm recombination detection (*13*). The sequences used corresponded to positions 2–7,407 in shzh00-1. Bootscanning was performed with a window size of 500 nt and step of 20 nt. Because of gaps in alignment, break points 211, 660, 3,756, 4,608, and 6,072 correspond to positions 207, 647, 3,555, 4,406, and 5,854 in shzh00-1, respectively. UTR, untranslated region; VP, viral protein.

Twenty-four enterovirus sequences, mainly from HEV-A group were retrieved from the National Center for Biotechnology Information website. Phylogenetic analysis**,** performed with the MEGA4 program (*12*), indicated that CA16 strains shzh00-1, shzh05-1, and GZ08 did not cluster with CA16-G10 (Figure 1, panel A), although they indeed belonged to HEV-A and clustered with EV71A (BrCr), EV71B (EV71/9/97/SHA89), and EV71C (S10862-SAR-98) (Table). Thus, the so-called CA16 full-length sequences (shzh00-1, shzh05-1, and GZ08) from China are distinct from CA16-G10 full-length sequences.

**Table:** Origin of sequences used for phylogenetic analysis of coxsackievirus strains, China, 2010*

Strain	Type	Sample date	Country	City or state	GenBank accession no.
G-10	CA16	1951	South Africa		U05876
High point	CA4	1948	United States	North Carolina	AY421762
BrCr	EV71A	1970	United States	California	U22521
UH1/PM/1997	EV71B	1997	Malaysia		AM396587
S10862-SAR-98	EV71C	1998	Malaysia		DQ341359
shzh00-1	CA16	2000	China	Shenzhen	AY790926
shzh05-1	CA16	2005	China	Shenzhen	EU262658
GZ08	CA16	2008 Jun	China	Guangzhou	FJ198212
changchun013	CA16	2010 Jun	China	Changchun	HQ450582
changchun016	CA16	2010 Jun	China	Changchun	HQ450581
changchun023	CA16	2010 Jun	China	Changchun	HQ450580
changchun024	CA16	2010 Jun	China	Changchun	HQ450579
changchun025	CA16	2010 Jun	China	Changchun	HQ450578
changchun028	CA16	2010 Jun	China	Changchun	HQ450577
changchun029	CA16	2010 Jun	China	Changchun	HQ450576
changchun032	CA16	2010 Jun	China	Changchun	HQ450575
changchun033	CA16	2010 Jun	China	Changchun	HQ450574
changchun074	CA16	2010 Jun	China	Changchun	HQ450573
changchun075	CA16	2010 Jun	China	Changchun	HQ450572
changchun083	CA16	2010 Jun	China	Changchun	HQ450571
changchun090	CA16	2010 Jun	China	Changchun	HQ450570
changchun097	CA16	2010 Jun	China	Changchun	HQ450569
changchun104	CA16	2010 Jun	China	Changchun	HQ450568
changchun105	CA16	2010 Jun	China	Changchun	HQ450567
changchun108	CA16	2010 Jun	China	Changchun	HQ450566
changchun115	CA16	2010 Jun	China	Changchun	HQ450565
hangzhou023	CA16	2010 Jun	China	Hangzhou	HQ450561
hangzhou042	CA16	2010 Jun	China	Hangzhou	HQ450563
hangzhou046	CA16	2010 Jun	China	Hangzhou	HQ450560
hangzhou056	CA16	2010 Jun	China	Hangzhou	HQ450559
hangzhou114	CA4	2010 Jun	China	Hangzhou	HQ450564
hangzhou212	CA16	2010 Jun	China	Hangzhou	HQ450562

*CA, coxsackievirus A; EV, enterovirus.

Because the shzh00-1, shzh05-1, and GZ08 sequences clustered within the HEV-A group and were determined to be CA16 on the basis of the VP1 region (data not shown), we examined these 3 sequences for evidence of recombination. Shzh00-1 was chosen as representative because it was isolated earlier. It has 7,410 nt in its genome, including the 5′ untranslated region (UTR) (1–745), structural protein (746–3331), and nonstructural proteins P2 (3332–5065) and P3 (5066–7327), with the rest of its genome as 3′ UTR (7328–7410). Bootscanning with a sliding window of 500 nt, overlapping by 20 nt, was performed with the SimPlot program (version 3.5.1) (*14*) to investigate the possibility of recombination within the shzh00-1 sequence. Various HEV-A sequences were used as reference sequences. The results indicated that the 5′ UTR of the shzh00-1 sequence had relatively high similarity to CA4 (Figure 1, panel B). The P1 (VP4, VP2, VP3, and VP1) region was more similar to that of CA16-G10. However, part of the P2/P3 region of the shzh00-1 sequence (2C, 3A, 3B, and 3C) had relatively high similarity to EV71A but not to CA16-G10 (Figure 1, panel B). Similar results were obtained for the shzh05-1 and GZ08 sequences (data not shown). Thus, shzh00-1, shzh05-1, and GZ08 are recombinant type A HEVs that contain CA4, CA16, and EV71.

To examine the circulation status of recombinant CA16 in China, we characterized 24 additional CA16-related sequences from HFMD patients from central (Hangzhou, Zhejiang Province) and northeastern (Changchun, Jilin Province) China (Table). The break point around position 3555 interested us most because it not only roughly separated the P1 region from P2/P3 but also divided the open reading frame (746–7327) into CA16-like and non–CA16-like fragments. Thus, we selected this region spanning the recombination break point between the CA16 sequence and the downstream sequence.

**Figure 2:**
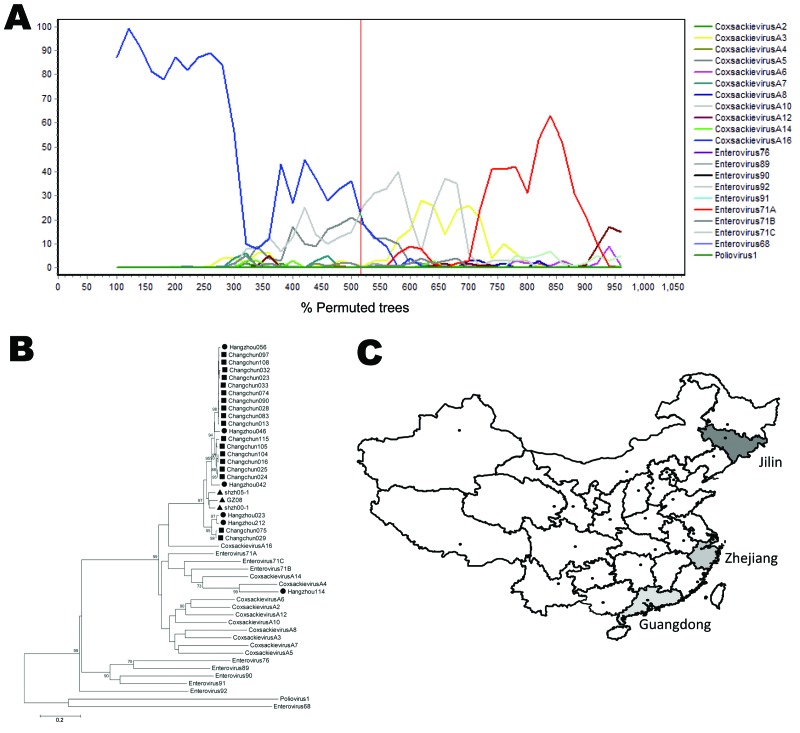
Phylogenetic analysis of Changchun and Hangzhou coxsackievirus 16A (CA16) sequences. A) Bootscanning results representing selected Changchun and Hangzhou sequences. changchun104 was shown for bootscanning analysis with human enterovirus A (HEV-A) sequences and shzh00-1 as references. The results suggested changchun104 was similar to shzh00-1. The red vertical line indicates position 3,555, which corresponded to shzh00-1. B) hangzhou212, hangzhou023, and all Changchun sequences clustered with shzh00-1, shzh05-1, and GZ08, with a very high bootstrap value (100%). Another Hangzhou sequence, hangzhou114, was most likely related to CA4. ● indicates Hangzhou sequences; ■ indicates Changchun sequences, and ▲ indicates shzh00-1, shzh05-1, and GZ08. Scale bar indicates nucleotide substitutions per site. C) Map of the People’s Republic of China indicating the provinces where shzh00-1–like CA16 sequences were characterized.

Bootscanning of these new sequences showed that the sequences from Changchun and Hangzhou have a recombination pattern similar to that of shzh00–1 (Figure 2, panel A; data not shown). Most Changchun and Hangzhou sequences changed from a CA16-like fragment into a non–CA16-like fragment at the break point around position 3555. However, when shzh00-1 was used as the reference sequence, bootscanning of these Changchun and Hangzhou sequences showed a high similarity to shzh00-1 through the entire sequenced region (data not shown). Phylogenetic analysis indicated that 23 of the Changchun and Hangzhou sequences formed a strong cluster with shzh00-1, shzh05-1, and GZ08 (Figure 2, panel B). Notably, 1 sequence clustered with the CA4 reference sequence, indicating that CA4-related viruses are also circulating among HFMD patients in China. These data suggest that shzh00-1 and these Changchun and Hangzhou sequences are likely derived from a common ancestor. New sequences identified in this study have been submitted to GenBank under accession nos. HQ450559–HQ450582.

## Conclusions

As many as 40% of cases of HFMD in China have been attributed to CA16 infection on the basis of partial viral genome determination (*11*). In the current study, we demonstrated that circulating CA16 viruses in China are actually complex recombinant viruses involving multiple type A HEVs, including CA4, CA16, and EV71 (Figure 1). The 5′ UTR region (207–647 bp) of these viruses had the highest similarity to CA4. Most of the P1 region resembled that of the prototype CA16-G10 strain. The nonstructural protein domains (P2 and P3) had a 1.5-kb fragment (4,406–5,854 bp) that was most similar to EV71A. Several regions of shzh00-1 remain unclassified, including part of 2A, 2B, part of 2C, and 3D.

Over the past 30 years, numerous large outbreaks of CA16-associated HFMD, along with outbreaks caused by EV71, have been reported in China. However, full molecular characterization of circulating CA16 viruses in China had not been conducted. The current study provides evidence that circulating recombinant forms of the CA16-related viruses are prevalent among HFMD patients throughout China (Figure 2, panel C). The origin, including the place and date, of the current recombinant CA16 viruses is not clear. The exact parental viral strains involved in the generation of CA16 recombinant viruses are also unknown. Some of the parental virus strains of China CA16 could have become extinct or are yet to be discovered. Notably, CA4 was detected in 1 of our samples from Zhejiang Province (central China) and has also been reported in Gansu Province (northwestern China) (*15*). The identification of CA16-related HFMD, based mainly on VP1 sequences, has been widely reported in many parts of China, including Guangdong, Fujian, Jiangsu, and Inner Mongolia Provinces, and Beijing and Shanghai. Unfortunately, the sequences of other parts of these viral genomes, especially the regions spanning the recombination site, have not been determined. Further detailed characterization of HEV sequences from HFMD patients is still needed, but the information obtained thus far has implications for addressing the future emergence of new pathogenic HEVs and for vaccine development to manage the increasing prevalence of HFMD.
